# CSE reduces OTUD4 triggering lung epithelial cell apoptosis via PAI-1 degradation

**DOI:** 10.1038/s41419-023-06131-1

**Published:** 2023-09-19

**Authors:** Lijuan Luo, Tiao Li, Zihang Zeng, Herui Li, Xue He, Yan Chen

**Affiliations:** 1 Department of Respiratory and Critical Care Medicine, The Second Xiangya Hospital of Central South University, Changsha, China; 2 Research Unit of Respiratory Disease, Central South University, Changsha, China; 3 Diagnosis and Treatment Center of Respiratory Disease, Central South University, Changsha, China

**Keywords:** Apoptosis, Ubiquitylation

## Abstract

Ovarian tumor family deubiquitinase 4 (OTUD4), a member of the OTU deubiquitinating enzyme, is implicated to decrease in cancer to regulate cell apoptosis. However, the role of OTUD4 in cigarette smoke induced epithelial cell apoptosis and its mechanism have not been elucidated. In this study, we showed that OTUD4 protein reduced in CSE treated mice and airway epithelial cells. OTUD4 silence aggravated cell apoptosis and emphysematous change in the lung tissue of cigarette smoke extract (CSE) treated mice. Additionally, restoration of OTUD4 in the lung of mice alleviated CSE induced apoptosis and emphysematous morphology change. The effect of OTUD4 on cell apoptosis was also confirmed in vitro. Through protein profile screening, we identified that OTUD4 may interact with plasminogen activator inhibitor 1(PAI-1). We further confirmed that OTUD4 interacted with PAI-1 for de-ubiquitination and inhibiting CSE induced PAI-1 degradation. Furthermore, the protective role of OTUD4 in airway epithelial cells apoptosis was blocked by PAI-1 deactivation. Taken together, our data suggest that OTUD4 regulates cigarette smoke (CS)-triggered airway epithelial cell apoptosis via modulating PAI-1 degradation. Targeting OUTD4/PAI-1 signaling might potentially provide a therapeutic target against the lung cell apoptosis in cigarette smoke (CS)-induced emphysema.

## Introduction

Chronic obstructive pulmonary disease (COPD) is one of the top three causes of death worldwide [1], which is characterized by irreversible airway obstruction, accelerated lung function decline, and a heterogeneous combination of bronchitis and emphysema [2]. Aberrant inflammatory, oxidation imbalance and cellular apoptosis in the respiratory system in response to cigarette smoke (CS) are considered as main pathogenesis of COPD [3]. CS exposure has been reported to cause various type of cell death including apoptosis, necrosis and ferroptosis [4, 5]. Previous studies demonstrated that bronchial epithelial cells and animal lungs exposed to cigarette smoke extract (CSE)/CS increased cellular apoptosis, thus promoted emphysema and the development of COPD [6–8].

Ubiquitination is an important post-translational modification that maintains proteostasis via proteasome or lysosome and regulates numerous cellular processes, including autophagy, DNA damage signaling, and inflammation [9, 10]. The ubiquitin ligases and deubiquitin enzymes (DUBs) interacted with substrates to regulate its ubiquitination. Thus, DUBs are likely to modulate cellular functions, including inflammation, cell circle arrest and cell death via targeting distinct substrates [11, 12]. Previous research has showed that CS destabilized the ubiquitin proteasome system (UPS) leading to DNA damage, cytotoxicity, and further emphysematous change [13]. What’s more, CS causes a significant accumulation of poly-ubiquitinated proteins in vivo and vitro [6, 14, 15]. However, the changes of DUBs under cigarette smoke exposure and their role in CS induced COPD have not been elucidated.

Ovarian tumor family deubiquitinase 4 (OTUD4) belongs to the ovarian tumor proteases (OTUs) DUB family and is implicated to cleaves K48- and K63- linked poly ubiquitin chains [16–19]. It regulated DNA repairment, inflammation, cell proliferation, and apoptosis by specific deubiquitylation [16, 17, 20, 21]. TLR-mediated activation of NF-κB is negatively regulated by OTUD4, and macrophages from OTUD4^−/−^ mice exhibit increased inflammatory signaling upon TLR stimulation [16]. What’s more, loss of OTUD4 in Hela cells impaired stress granule formation and promoted apoptosis via activating caspase 3 [20]. In addition, a meta-analysis of GWAS studies reported that OTUD4 is associated with disease susceptibility in COPD [22]. However, whether OTUD4 is involved in smoking induced airway epithelial cell apoptosis and participates in COPD pathogenesis is interested to be investigated.

Plasminogen activator inhibitor 1 (PAI-1) is a multifunctional protein that belongs to the SERPIN superfamily of serine protease inhibitors, also known as serpine1 [23]. It is an effective inhibitor of plasminogen activators, particularly urokinase-plasminogen activator (uPA) and tissue-type plasminogen activator and binds to the LDL receptor (LRP) and vitronectin [24]. PAI-1 is involved in cell physiological activities, including cell adhesion, proliferation, migration, and viability [25]. Previous study demonstrated an antiapoptotic role for PAI-1, decreased expression of PAI-1 promotes cell apoptosis and inhibit cell proliferation [26]. Enhanced apoptosis was present in the lungs of PAI-1^-/-^ mice compare with wild-type mice, which contribute to LPS-induced acute lung injury [27].

In this study, we identified that the decreased OTUD4 exaggerated apoptosis in the lung epithelia via increasing PAI-1 ubiquitination and degradation. Restoration of OTUD4 in mice mitigated lung cell apoptosis, thus contributed to the alleviation of emphysema morphology change in COPD. Above all, this study added preliminary evidence that CSE induced OTUD4 reduction promoted epithelia apoptosis in COPD pathogenesis.

## Materials and methods

### Human samples

Peripheral lung tissues from subjects who have been proceed with thoracic surgery at the Department of Thoracic Surgery, Second Xiangya Hospital of Central South University. COPD patients were previously diagnosed according to Global Initiative for Chronic Obstructive Lung Disease (forced expiratory volume in 1 s/forced vital capacity [FEV1/FVC] <0.7) [28]. The study was approved by the Medical Ethics Committee of the Second Xiangya Hospital of Central South University (No. 2021733). Written informed consent was obtained from all human subjects before their enrollment into the study, which was conducted in accordance with the Declaration of Helsinki.

### Preparation of CSE

One full-strength cigarette (Marlboro; Longyan Tobacco Industrial Co, Ltd, Fujian, China; tar: 10 mg, nicotine: 1.1 mg, carbon monoxide: 11 mg) were combusted using a modified syringe-driven apparatus. 1 cigarette were collected in 10 mL Dulbecco’s minimum essential media (DMEM) for cell experiments. The smoke was bubbled into DMEM then filtered through a 0.2 μm pore-size filter to sterilize and remove particulate matter and was used immediately. The 100% CSE sample was thereafter diluted with DMEM to appropriate percentages of CSE solution. CSE was freshly prepared for every experiment.

### Animal procedure

The emphysema mouse model was built according to the protocol of Zhang et al. with slight modification [29]. Six-week-old, specific pathogen-free, BALB/c mice (21–23 g each) (Slyke Jingda, Hunan, China) were injected intraperitoneally with CSE or PBS at days 0, 11, and 22 (0.3 mL per injection). OTUD4 overexpress lentivirus, OTUD4 silent lentivirus (10^9^ ifu/mL, GeneChem, Shanghai, China) or PBS (0.1 mL) was instilled intratracheally at day 14. Tiplaxtinin (5 mg/kg,100 μL diluted in DMSO, S7922, Selleck Chemicals, Texas, USA) was administered via oral gavage daily during mouse modeling [30]. Mice were randomly assigned to different groups for experimental treatment by a free online randomization tool (www.graphpad.com/quickcalcs/randomize1). The animals were sacrificed on day 28. The results were blindly analyzed by investigators. All animal care and experimental protocols were approved by the Animal Care and Use Committee of the Second Xiangya Hospital of Central South University (No. 2021789).

### Lung tissue morphometry and immunohistochemistry (IHC)

Lung tissue samples were fixed in 4% formaldehyde, cut into 3.5 mm-thick sections, and stained with hematoxylin and eosin (HE). The morphology of lung tissues of mice was observed in random fields by light microscopy. The mean linear intercept (MLI) and destructive index (DI) were measured at a magnification of 100× as previously described [29]. About IHC, the slides were incubated with anti-OTUD4 (1:3000, A304–605A, BETHYL). Quantitative measurements of OTUD4 positive cells in the lung tissue were performed according to previously described methods [31]. Briefly, OTUD4-positive and -negative cells were counted in each specimen. The cells positive for the OTUD4 antibody staining were expressed as a percentage of total cells. Sections were examined using a light microscope.

### TUNEL assay

The TUNEL assay was analyzed according to apoptosis detection kit (Cat: # G1504, Wuhan Saiwell Biotechnology Co., Ltd, Wuhan, China). Briefly, the sections of lung tissue or cells climbing were deparaffinized in xylene and rehydrated in PBS buffer. The sections were incubated with 15 μg/mL proteinase K for 20 min at 37 °C. After washing three times with PBS buffer, the sections were incubated in 1× equilibration buffer for 30 min, then incubated with a mixture containing 50 μL of Biotin‐dUTP Labeling Mix and 3 μL of TdT Enzyme for 1 h at 37 °C in the dark. Then, the slides were incubated with 100 μL stopping buffer for 10 min and then rinsed in PBS three times. The specimen was covered in Streptavidin‐HRP for 30 min and washed in PBS three times. The slides were visualized by DAB substrate and observed by microscope. TUNEL‐positive cells were counted, and the apoptotic index was calculated as a ratio of (apoptotic cell number)/ (total cell number) in each field under magnifying view of 200x.

### Cells and reagents

Airway epithelial cells BEAS-2b and HBE were purchased from the Chinese Academy of Sciences (Shanghai, China) and cultured in DMEM (Hyclone, Logan, UT, USA) supplemented with 10% fetal bovine serum and 50 U/mL penicillin and streptomycin (Gibco, Thermo Fisher Scientific, Waltham, MA, USA) at 37 °C in a 5% CO_2_ culture chamber. Lung epithelial A549 cells were cultured in RPMI-1640 medium supplemented with 10% fetal bovine serum and 50 U/mL penicillin and streptomycin (Gibco, Thermo Fisher Scientific, Waltham, MA, USA) at 37 °C in a 5% CO_2_ culture chamber. Starvation for 24 h was performed before exposure to CSE, siRNA, and/or plasmids. OTUD4 antibody (Cat: #A304–605A) was form Bethyl company (Montgomery, USA), PAI-1(Cat: sc-5297) was from Santa Cruz Biotechnology (Shanghai, China), Bax antibody (Cat: 50599-2-Ig) was from proteintech (Rosemont, USA). BCL2 antibody (Cat: #3498) and cleaved caspase3 antibody (Cat: #9664) are from CST company (Massachusetts, USA). Lipofectamine 3000 (Cat: #L3000008) were purchased from Thermo Fisher Scientific (Waltham, MA, USA), Cycloheximide (CHX, HY-12320) and MG132 (HY-13259) were purchased from MedChemExpress (New Jersey, USA). Tiplaxtinin (S7922) was purchased from Selleck Chemicals (Texas, USA).

### Protein identification by mass spectrometry

Spectra of the peptide pools were obtained on a MALDI-TOF/TOF instrument (ABI4700; Applied Biosystems). MALDI mass spectra (∼100) were acquired for each digest and internally calibrated using trypsin autolysis peaks, and the top seven most abundant signals (within m/z 900–2,000) were automatically selected for tandem analysis. Precursor masses corresponding to a list of contaminant masses commonly observed in this laboratory were excluded from the list of precursor masses for tandem analysis. The peptide fragmentation spectra were processed using Data Explorer (version 4.5; Applied Biosystems). After centroiding and background subtraction, the spectra were used to search the National Center for Biotechnology Information nonredundant database using MASCOT (version 1.9; Matrix Sciences).

### Small-interfering RNA transfection

Scramble siRNA or OTUD4 siRNA were constructed and purchased from RiboBio company (Guangzhou, China). BEAS-2b cells were transfected with scramble or OTUD4 siRNA for 48–72 h following the manufacturer’s instructions. Knockdown of OTUD4 with siRNA were confirmed by RT-qPCR and western blotting. The OTUD4 siRNA used in the experiments was as follows: GTAGCTGATGAAGATAACA.

### Plasmid transfection

The plasmid vector GV362, OTUD4 overexpressed plasmids, PAI-1 overexpressed plasmids and ub-related plasmid were from GeneChem company (Shanghai, China). Briefly, 2 × 10^5^ of BEAS-2b cells or HEK293T cells were used for each transfection. 2 μg of plasmids were transfected into BEAS-2b cells or HEK293T cells with lipofectamine 3000 for 48 h according to the manufacturer’s instructions.

### Western blot analysis

After stimulation, whole cell extracts were prepared using RIPA buffer [19]. Cell lysates were separated with SDS-PAGE and transferred to PVDF membrane. Then, PVDF membranes were incubated with OTUD4 antibody (1:3000), Bax antibody (1:1000), BCL2 antibody (1:1000), cleaved caspase 3 antibody (1:1000) and PAI-1 antibody (1:200) overnight. To standardize the expression of each protein, the membranes were reported with anti-β-actin antibody (1:5000). The membranes were then incubated with the appropriate peroxidase-conjugated secondary antibodies (1:5000, SA00001-1/SA00001-2, proteintech). The bound antibodies were visualized by chemiluminescence (ECL plus; GE Healthcare, Buckingham, UK).

### Isolation of RNA and real-time qPCR

The total RNA was isolated from the cells using TRIZOL reagent (Invitrogen, California, USA) and then treated with DNase I (Invitrogen, California, USA) according to the manufacturer’s instructions. Total RNA was then reverse transcribed using the Revert Aid First Strand cDNA Synthesis Kit (Thermo Scientific, Massachusetts, USA). Subsequently, real-time PCR was performed using a Step One Plus real-time PCR system (Life Technologies, Carlsbad, CA, USA). GAPDH was used as an internal control. The sequences of all primers used were:

OTUD4-F: 5′-AGACCCGAACCAAGCACAT-3′.

OTUD4-R: 5′-CTGGCTTTTGTTCCGCA-3′.

GAPDH-F: 5′-TCAAGAAGGTGGTGAAGCAG-3′.

GAPDH-R: 5′-CGTCAAAGGTGGAGGAGTG-3′.

Transcript levels were normalized to the housekeeping gene GAPDH levels. The relative mRNA levels were calculated according to the comparative Ct (^ΔΔ^Ct) method, where Ct represents the threshold cycle for each transcript.

### Co-immunoprecipitation

Co-immunoprecipitation (Co-IP) was conducted as previously described [32]. Briefly, 1 mg of cell lysates (in PBS with 0.5% Tween 20, and protease inhibitors) were incubated with specific primary antibodies for overnight at 4 °C. The mixture was added to 40 μL of protein A/G-agaroses for an additional 2 h at 4 °C. The precipitated complex was washed three times with 0.5% Tween 20 in PBS and analyzed by immunoblotting as described above.

### Ubiquitination assay

The BEAS-2b cells or HEK293T cells were co-transfected with plasmid. After incubation with 5 μM MG132 for 5 h before harvesting, and then which they were washed with PBS and lysed with IP lysis/wash buffer with protease inhibitor and phosphatase inhibitor (Roche) and 10 μM N-ethylmaleimide (NEM; Sigma, St Louis, MO, USA) on ice for 30 min. The cleared lysates were quantified, and an equal amount of each lysate was used for immunoprecipitation with protein A/G agarose (Sigma) pre-bound with the specified antibodies. The resin beads were washed with lysis buffer, and samples were eluted. The eluted fraction was further separated on an SDS-PAGE gel. Subsequent western blotting was performed using the indicated antibodies using the enhanced chemiluminescence-detection system (BIO-RAD, CA, USA).

### Immunofluorescence staining assays

Immunofluorescence (IF) staining was conducted as previously described [33]. Briefly, cells (2 × 10^5^) were plated at 70% confluence onto 35 mm MatTek glass-bottomed culture dishes. Immunofluorescent cell imaging was performed with a Nikon A1 confocal microscope. Cells were washed with PBS and fixed with 4% paraformaldehyde for 20 min, then exposed to 15% BSA, 1:500 dilutions of primary antibodies, and 1:1000 dilutions of Alexa-488- or Alexa-647-labeled goat anti-mouse or anti-rabbit secondary antibodies sequentially for immunostaining.

### Statistical analysis

All of the statistical tests were performed using SPSS 23.0. The data are presented as mean ± SD, or as counts or percentages as relevant. Two-group comparisons were performed using an independent sample t-test. Multiple groups were compared using one-way ANOVA. Statistical significance is denoted by *p* < 0.05.

## Results

### OTUD4 is reduced in the lung tissue of COPD patients and emphysema mouse model

**Fig. 1:**
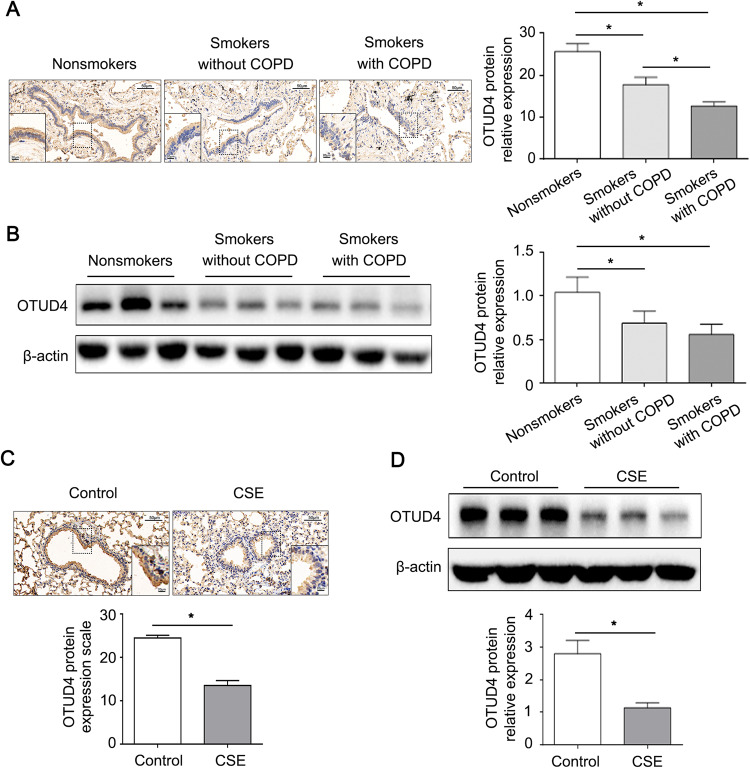
OTUD4 is downregulated in the lung tissue of COPD patients and CSE-induced emphysema mouse model. **A** Representative result of IHC (400× magnification) for OTUD4 in human lung tissue. **B** Western blotting to detect OTUD4 protein expression in human lung tissue. **C** OTUD4 was detected with IHC in the lung tissue of mice model. **D** Western blotting was applied to detect the OTUD4 protein expression in the lung tissue of mice. Data represents as mean ± SD of three independent experiments. ^*^P < 0.05.

Previous research proved that DUBs are regulated by cigarette smoke [34, 35]. We firstly performed IHC to detect the expression of OTUD4 in the peripheral lung tissue specimens from nonsmokers, smokers without COPD, and smokers with COPD. The results showed that OTUD4 was widely distributed in the airway and lung epithelium, and its abundance was lower in the smoker patients with or without COPD than nonsmokers (Fig. 1A).

To confirm these results, the western blot analysis was applied and demonstrated that OTUD4 protein levels were significantly reduced in smokers with and without COPD comparing to nonsmokers (Fig. 1B). Interestingly, the OTUD4 expression was obvious reduced in the lung tissue of cigarette smoke extract induced emphysema mouse model (Fig. 1C, D). These results indicated that OTUD4 protein levels were decreased significantly in cigarette smoke-induced COPD patients and emphysema mouse model.

### OTUD4 deficiency aggravates airway epithelium apoptosis in CSE induced emphysema

**Fig. 2:**
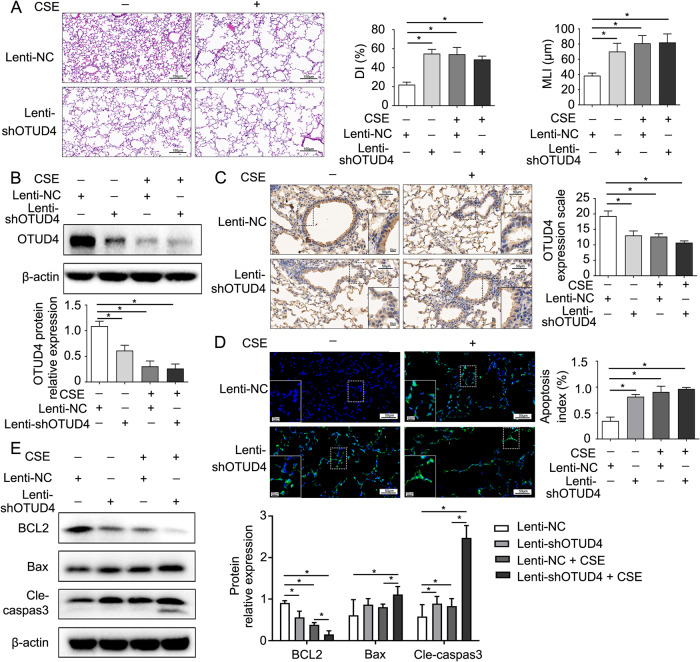
OTUD4 deficiency accelerates CSE induced emphysema via increasing airway epithelium apoptosis. **A** Representative HE staining images (200×) of lung tissue were from control mice and OTUD4 deficiency mice. DI (%) and MLI (μm) were measured. Immunoblotting (**B**) and IHC (**C**) were used to detect OTUD4 protein expression (400×). **D** Apoptosis of lung cells in indicated group of mice were detected via TUNEL assay (400×). Apoptosis index (apoptotic cells/ total cells, %) was calculated. **E** BCL2, Bax and cleaved-caspase 3 protein expression was detected via immunoblotting. Lenti-NC: negative control; Lenti-shOTUD4: OTUD4 knockdown. Data were shown as mean ± SD of three independent experiments. ^*^P < 0.05.

OTUD4 is reported to regulates cell death [20, 21]. To investigate the potential role of OTUD4 in the lung of mice, a CSE mediated experimental emphysema model was established with the previous described methods. HE staining exhibited that enlargement of alveolar airspaces and destruction of lung parenchyma were presented in OTUD4 knockdown and CSE treated mice. Furthermore, the morphological indexes of emphysema including MLI and DI were dramatically increased following the reduction of OTUD4 (Fig. 2A). We found that the OTUD4 protein levels detected by western blot and IHC were decreased significantly in CSE mouse model comparing to controls (Fig. 2B, C). Interesting, in situ TUNEL assay confirmed that lack of OTUD4 enhanced the cell apoptosis in the lung tissue of CSE induced emphysema model (Fig. 2D). Besides, OTUD4 knockdown increased pro-apoptotic protein Bax and Cleaved caspase 3 expression, while vigorously reduced anti-apoptotic BCL2 protein expression in lung tissue (Fig. 2E). These data illustrated that OTUD4 reduction facilitate CSE-induced emphysema in mice by increasing lung cell apoptosis.

### Enhanced OTUD4 alleviates emphysema by reducing epithelia apoptosis

**Fig. 3:**
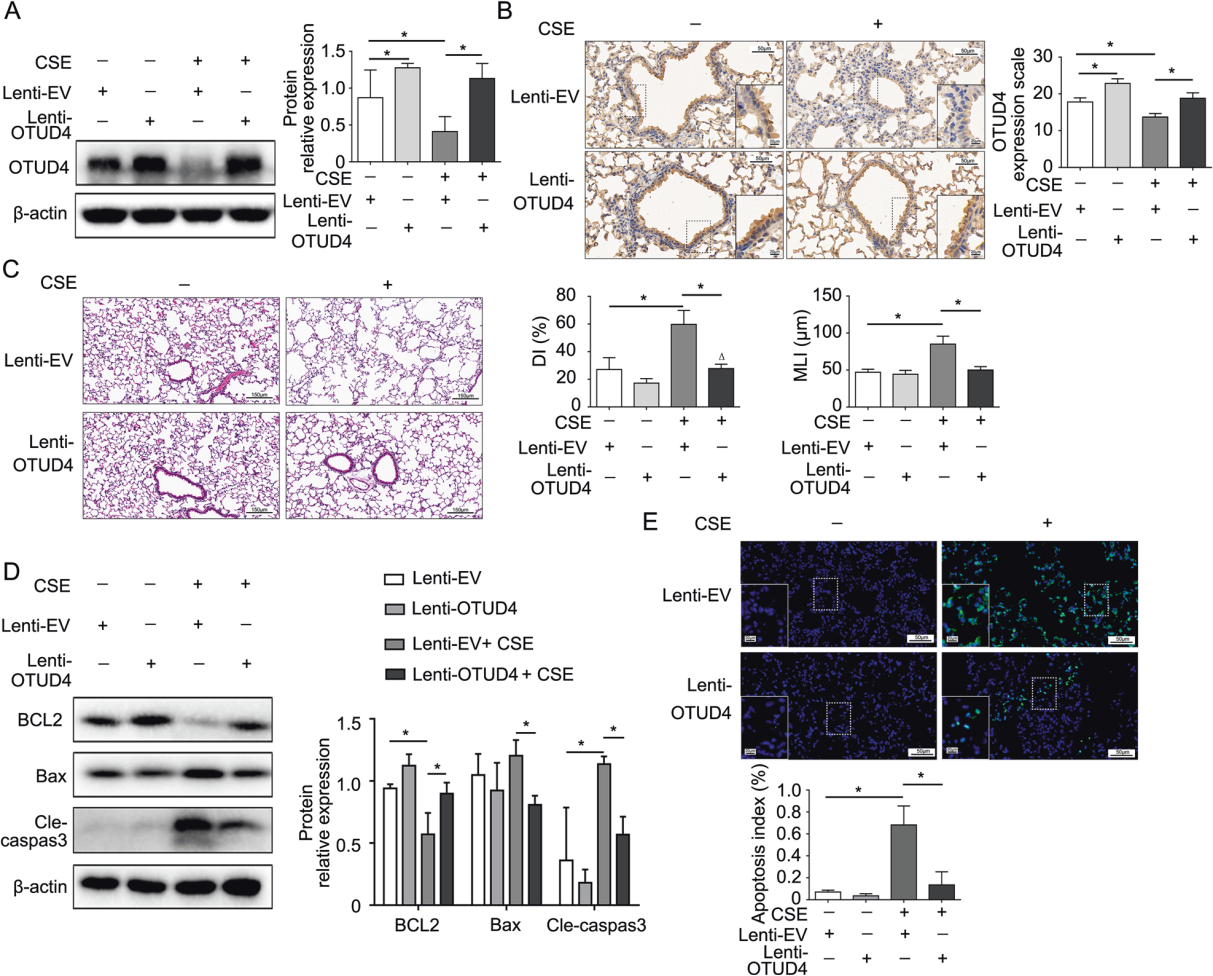
Enhanced OTUD4 alleviates emphysema by reducing cell apoptosis. **A** Western blot to detect the expression of OTUD4 protein in the lung tissue of mice. **B** Representative results of IHC (400×) for OTUD4 in different groups were shown. **C** Histological changes of lung sections were shown with H&E (200×) staining. Morphometric measurements of MLI (μm) and DI (%) were plotted. **D** The expression of apoptotic protein including BCL2, Bax and cleaved caspase 3 were detected in the lung tissue of mice. **E** Apoptotic nuclei were detected by TUNEL staining (400×) in the lung tissue of mice. Lenti-EV: empty vector, Lenti-OTUD4: OTUD4 overexpression; Data were shown as mean ± SD of three independent experiments. ^*^P < 0.05.

OTUD4 decreased in the murine lung tissue after CSE treatment and aggravated cell apoptosis. However, whether restoring the OTUD4 could protect the mice from emphysema damage is not clear. We applied OTUD4 lentivirus intratracheally to overexpress OTUD4 in the lung of CSE treated mice (Fig. 3A, B). HE staining showed that enhanced OTUD4 expression partially reversed the enlargement of alveolar airspaces and destruction of lung parenchyma after CSE treatment (Fig. 3C, upper panel).

What’s more, the morphological indexes of emphysema including MLI and DI were decreased after OTUD4 upregulation (Fig. 3C, lower two panels). Contrary to reduced OTUD4 expression, we proved that overexpression of OTUD4 reduced Bax and cleaved caspase 3 expression and partially reversed the anti-apoptotic protein BCL2 expression (Fig. 3D). We further confirmed that increased OTUD4 prevented apoptosis in the lung tissue of CSE mouse model through TUNEL assay (Fig. 3E). Overall, these findings revealed that OTUD4 play a potential role in protecting CSE- induced emphysema in mice by decreasing cell apoptosis.

### OTUD4 protein but not mRNA decreases in CSE treated lung epithelial cells

**Fig. 4:**
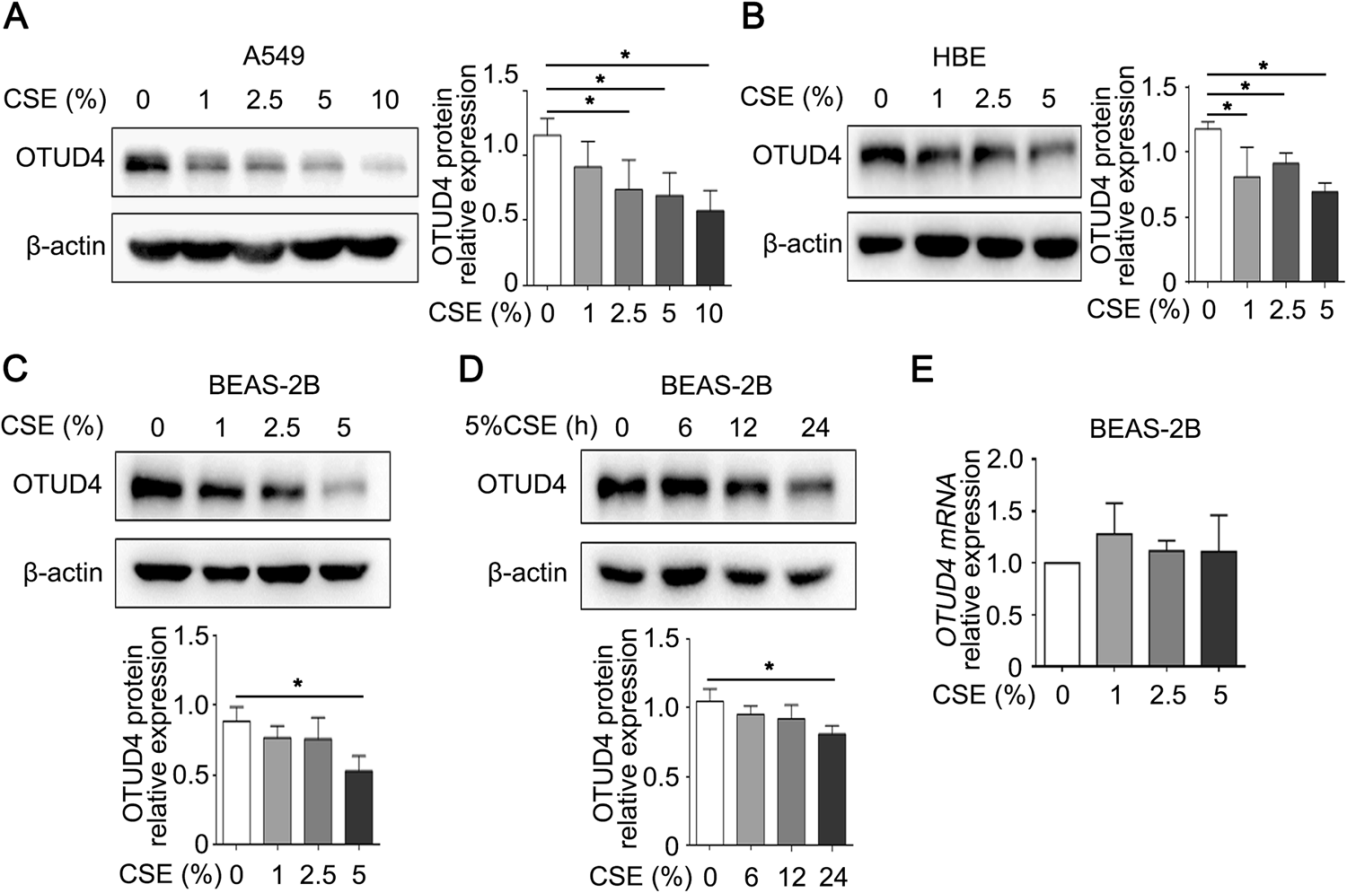
OTUD4 decreases in CSE induced lung epithelial cells. **A**–**D** OTUD4 relative expression was assayed by western blotting in type II epithelial A549 cells, HBEs and BEAS-2b. **E** RT-qPCR to detect the mRNA level of OTUD4 in CSE treated BEAS-2b cells. Data represents as mean ± SD of three independent experiments. ^*^P < 0.05.

From above results, OTUD4 protein is mainly expressed in the airway epithelium. In order to assess the OTUD4 level under cigarette smoke treat in vitro, we analyzed the protein expression of OTUD4 in the airway and lung epithelial cells exposed to CSE. OTUD4 decreased in both A549 and HBE cell lines with a CSE dose-dependent way (Fig. 4A, B). Besides, the reduction of OTUD4 protein was further discovered in a CSE dose-dependent and time-dependent manner in the BEAS-2b cells (Fig. 4C, D). Interestingly, the mRNA level did not differ in the BEAS-2b cells exposed to different concentrations of CSE (Fig. 4E). These results indicated that the protein and mRNA expression of OTUD4 in CSE treatment is discordant.

### OTUD4 regulates the apoptosis of lung epithelial cells

**Fig. 5:**
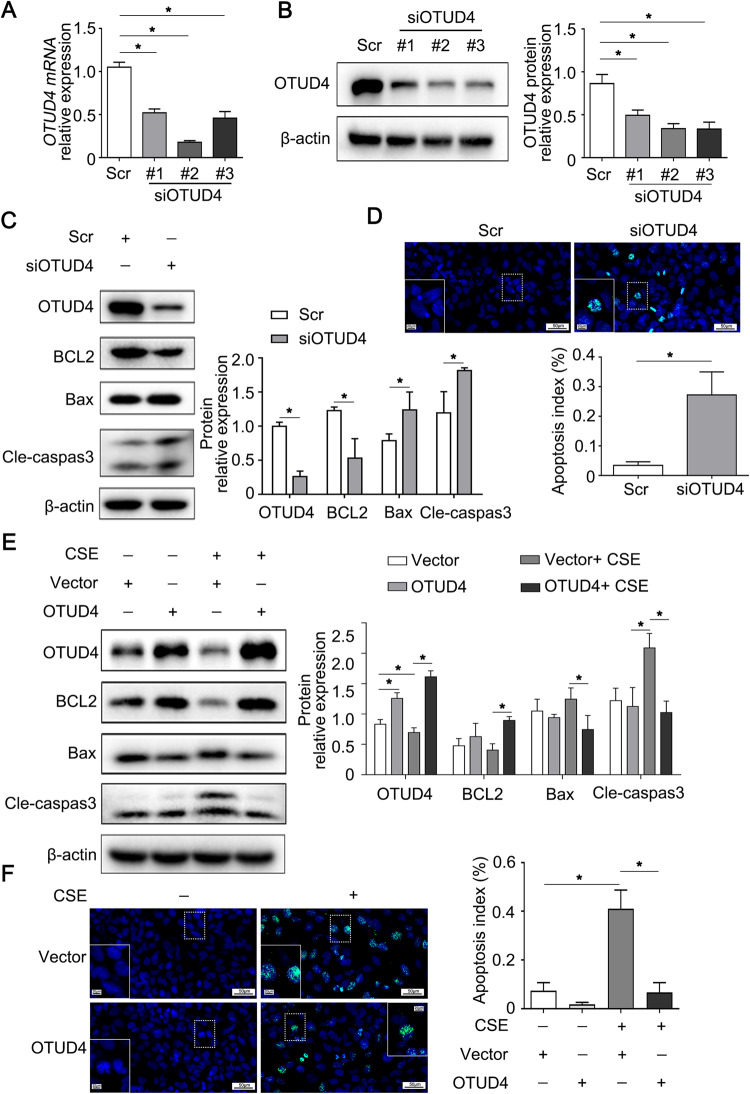
OTUD4 regulates apoptosis in lung epithelial cells induced by CSE. **A**, **B** The mRNA and protein level of OTUD4 were confirmed with RT-qPCR and western blot. **C** OTUD4, BCL2, Bax, cleaved-caspase 3 or β-actin were assayed with western blotting after OTUD4 knockdown. **D** TUNEL staining (400×) to detect apoptotic nuclei of BEAS-2b cells. **E** Western blot to detect the protein level of BCL2, Bax and cleaved caspase 3. **F** Apoptotic nuclei of BEAS-2b cells were detected by TUNEL staining (400×). Data were shown as mean ± SD of three independent experiments. *p < 0.05.

To confirm the above results, we next manipulate OTUD4 in BEAS-2b bronchial epithelial cells. Three strands of siRNAs targeting OTUD4 were applied to BEAS-2b cells. The second one reduced the mRNA and protein level of OTUD4 mostly, which was chosen for subsequent experiments (Fig. 5A, B). We firstly check the apoptotic protein after silence of OTUD4. As expected, we found that lack of OTUD4 also increased the pro-apoptotic protein of Bax, cleaved caspase 3 and severely decreased the anti-apoptotic protein of BCL2 in cells (Fig. 5C). In addition, we proved that knockdown of OTUD4 enhanced cell apoptosis in BEAS-2b through the TUNEL assay (Fig. 5D). Above results further demonstrated that OTUD4 silence promoted lung epithelial cell apoptosis.

To verify whether OTUD4 could prevent the epithelia apoptosis, we overexpressed OTUD4 in BEAS-2b cells exposed to CSE. We found that Bax and cleaved caspase 3 were reduced, while BCL2 was enhanced after CSE treatment (Fig. 5E). Furthermore, TUNEL- fluorescence assay showed that the apoptosis of BEAS-2b was significantly increased under CSE stimulation. While enhanced expression of OTUD4 obviously alleviated the epithelia apoptosis after CSE exposure (Fig. 5F). As expected, these results also demonstrated that OTUD4 prevents CSE-induced apoptosis in epithelial cells.

### OTUD4 deubiquitinates PAI-1 and inhibits its proteasomal degradation

**Fig. 6:**
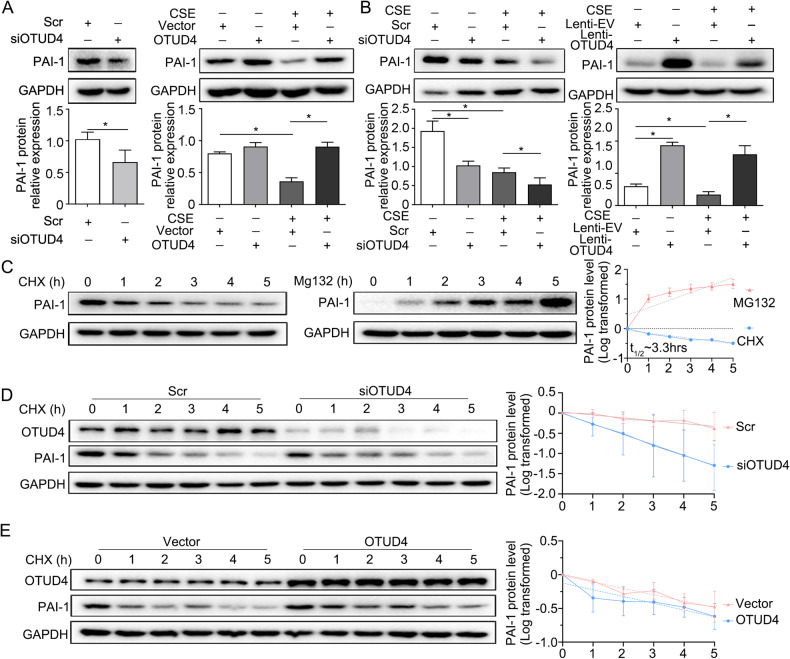
OTUD4 deubiquitinates PAI-1 and inhibits its proteasomal degradation. **A** Western-blot assayed the protein level of PAI-1 in BEAS-2b cells. **B** PAI-1 protein expression in the lung tissue of mice was detected by western blotting. **C** CHX or MG132 was subjected to BEAS-2b cells. PAI-1 is with a half-life of about 3.3 h. **D**, **E** PAI-1 protein expression was detected by western blotting in BEAS-2b cells after OTUD4 knockdown or overexpression with CHX treatment. Data represents as mean ± SD of three independent experiments. ^*^P < 0.05.

How the OTUD4 regulating apoptosis is still not clear. Through protein profile screening, we found that OTUD4 may interact with protein PAI-1. Reduced OTUD4 inhibited the expression of PAI-1 protein (Fig. 6A, left panel), while overexpression of OTUD4 partially reversed the protein level of PAI-1 in CSE treated BEAS-2b cells (Fig. 6A, right panel). The same results were confirmed in the lung tissue of CSE induced emphysema mice model (Fig. 6B). Considering that OTUD4 is a deubiquitinating enzyme, we use proteasome inhibitors MG132 and protein synthesis inhibitor chlorhexidine (CHX) respectively to detected whether PAI-1 is regulated by the ubiquitin proteasome pathway. CHX-treated cells showed that PAI-1 is unstable with a half-life of approximate 3.3 h (Fig. 6C, left and right panel). In addition, PAI-1 proteins tended to increase under MG132 treatment (Fig. 6C, middle panel). This data suggested that PAI-1 was degraded via proteasome mechanism. Next, we performed CHX and MG132 in the BEAS-2b cells after modulating of OTUD4. Results showed that OTUD4 knockdown promoted PAI-1 degradation, while OTUD4 overexpression slowdown the deduction of PAI-1 expression (Fig. 6D, E).

**Fig. 7:**
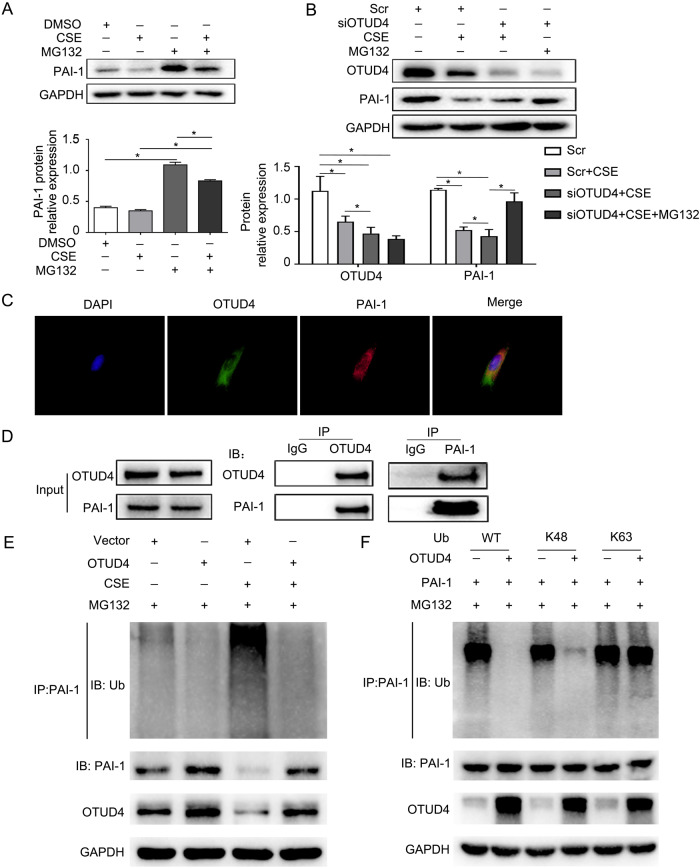
OTUD4 deubiquitinates PAI-1 and inhibits its proteasomal degradation. **A**, **B** BEAS-2b cells were treated with CSE or MG132, CSE or MG132 was subjected to BEAS-2b cells after OTUD4 knockdown, Western blotting was used to detect OTUD4 and PAI-1 protein level. **C** Immunofluorescence stanning (400×) of OTUD4 (green), PAI-1 (red) and DAPI (blue) and merged image (upper panel). D Immunoprecipitation of OTUD4 and PAI-1 (lower panel). **E** Ubiquitination assay of PAI-1 in CSE induced BEAS-2b cells after OTUD4 overexpression. **F** WT, K48, or K63 Ub was co-transfected with PAI-1 and OTUD4 into HEK293T cells. After treatment with 5 μM MG132 for 5 h, cell lysates were subjected to ubiquitination assay. Data represents as mean ± SD of three independent experiments. ^*^P < 0.05.

To understand if CSE-induced PAI-1 reduction is mediated by proteasome, we applied CSE and MG132 to cells, which found that MG132 treatment rescued the PAI-1 expression in CSE treated cell (Fig. 7A). What’s more, OTUD4 silence contributed to the instability of PAI-1 induced by CSE (Fig. 7B). Further immunofluorescent stanning and co-immunoprecipitation proved that OTUD4 was associated with PAI-1 (Fig. 7C, D). In addition, ubiquitination assay showed that CSE can increase ubiquitination and degradation of PAI-1, while enhanced OTUD4 expression can decrease the ubiquitination level of PAI-1 induced by CSE (Fig. 7E). And then to identify which type of ubiquitin chain of PAI-1 was affected by OTUD4, we co-transfected HEK293T cells with PAI-1, OTUD4, WT ubiquitin, K48-specific or K63-specific ubiquitin plasmid. Our results indicated that OTUD4 could efficiently remove K48-linked ubiquitin chain from PAI-1(Fig. 7F). Taken together, OTUD4 is a specific DUB responsible for PAI-1 deubiquitination and stabilization.

### Inhibition of PAI-1 perturbs the protective effect of OTUD4 on CSE induced apoptosis

**Fig. 8:**
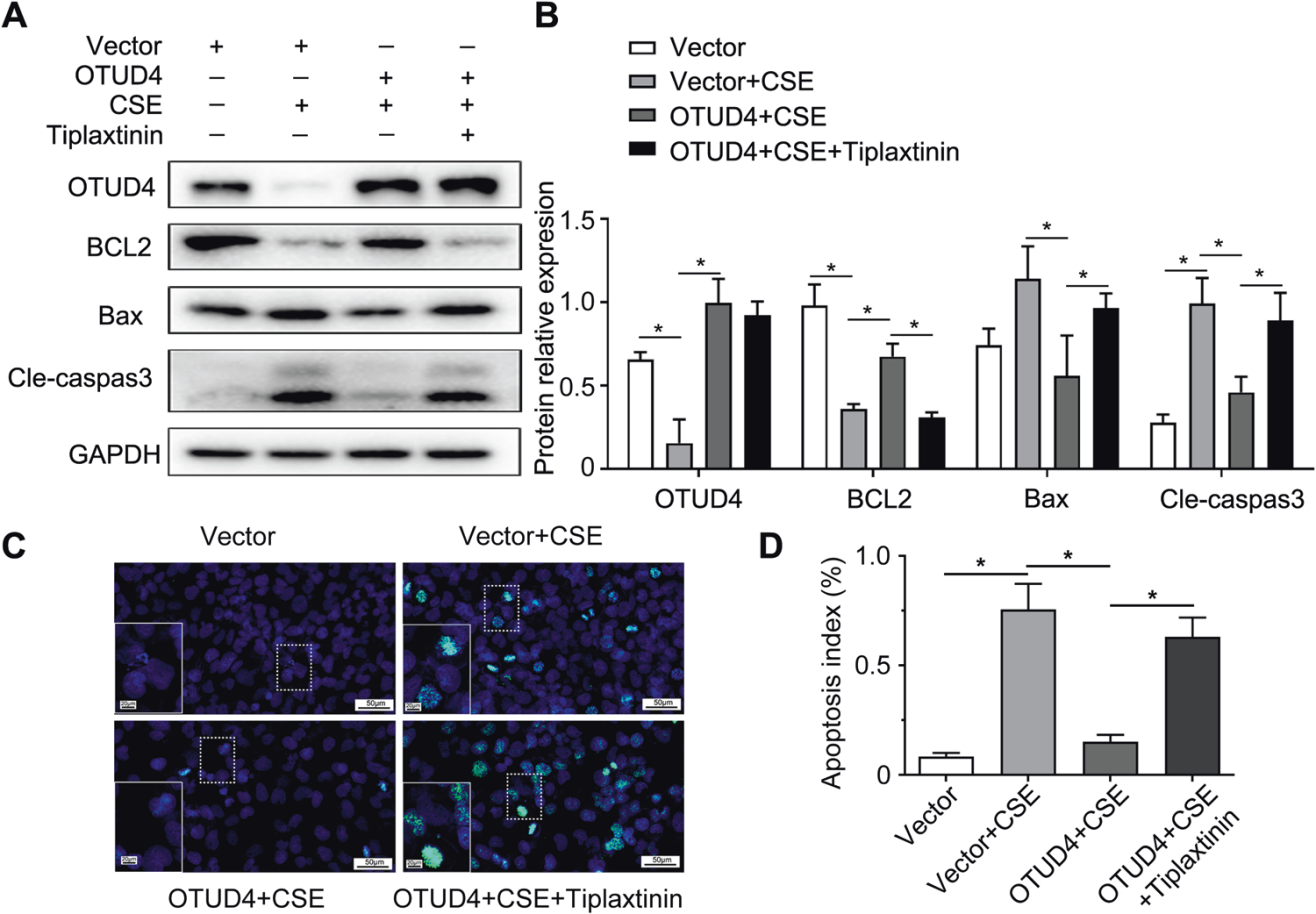
Inhibition of PAI-1 perturbs the protective effect of OTUD4 on CSE induced apoptosis in BEAS-2b cells. **A** OTUD4 overexpressed cells were treated with PAI-1 inhibitor (tiplaxitinin) or CSE. OTUD4, BCL2, Bax, and Cleaved-caspase3 were detected by western blot. **B** Densitometry of OTUD4, BCL2, Bax, Cleaved-caspase3. **C** Apoptotic nuclei were detected by TUNEL staining (400×) in OTUD4 overexpressed BEAS-2b cells treated with CSE or tiplaxitinin. **D** Apoptotic index (%) was plotted. Data represents as mean ± SD of three independent experiments. ^*^P < 0.05.

In order to further prove that the protection of OTUD4 from CSE-induced apoptosis is achieved by regulating PAI-1, we used PAI-1 inhibitors, tiplaxtinin, to treat BEAS-2b cells with OTUD4 overexpression. Results showed that enhanced OTUD4 increased the expression of anti-apoptotic protein BCL2 and reduced the expression of pro-apoptotic proteins Bax and cleaved caspase3 in CSE-induced BEAS-2b cells. However, the addition of tiplaxtinin reversed the protective effect of OTUD4 and increased CSE induced cell apoptosis (Fig. 8A, B). What’s more, TUNEL assay showed that apoptotic cells were decreased significantly under OTUD4 overexpression, while obviously enhanced in CSE + OTUD4+tiplaxtinin group (Fig. 8C, D).

**Fig. 9:**
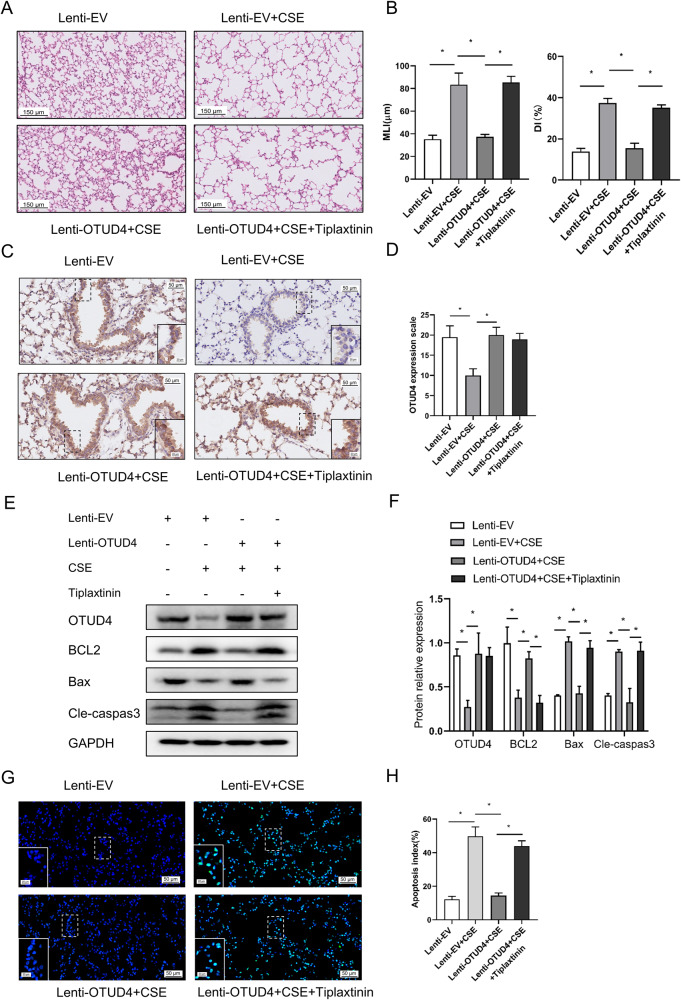
Inhibition of PAI-1 perturbs the protective effect of OTUD4 on CSE induced apoptosis in lung tissue of mice. **A**, **B** Histological changes of lung sections were shown with H&E (200×) staining. Morphometric measurements of MLI (μm) and DI (%) were plotted. **C-D** Representative results of IHC (400×) for OTUD4 in different groups were shown. **E**, **F** Western blot to detect the expression of OTUD4, BCL2, Bax and cleaved caspase 3 protein in the lung tissue of mice. **G**, **H** Apoptotic nuclei were detected by TUNEL staining (400×) in the lung tissue of mice. Lenti-EV: empty vector, Lenti-OTUD4: OTUD4 overexpression; Data were shown as mean ± SD of three independent experiments. ^*^P < 0.05.

In addition, we further confirmed these results through in vivo experiments. We performed administration of tiplaxtinin via oral gavage daily in mice with overexpression of OTUD4 under CSE intervention. We found that enhanced OTUD4 expression can improved the CSE induced emphysematous change, which include enlargement of alveolar airspaces, destruction of lung parenchyma and elevated morphological index MLI and DI. However, the treatment of tiplaxtinin can inhibit the protective effect of OTUD4 in CSE induced emphysema (Fig. 9A, B). We confirm that OTUD4 was successfully overexpressed in CSE induced emphysema mice (Fig. 9C, D). The results showed that overexpressed OTUD4 can increased the level of anti-apoptotic protein BCL2 and reduced the level of pro-apoptotic proteins Bax and cleaved caspase3 in the lung tissue of CSE induced emphysema mice. But there demonstrated opposite alternation in Lenti-OTUD4 + CSE+Tiplaxtinin group (Fig. 9E, F). Furthermore, TUNEL detection also confirmed that overexpression of OTUD4 can significantly reduce CSE induced lung cell apoptosis, while the apoptotic cells were significantly increased with tiplaxtinin treatment (Fig. 9G, H). All above inferred that OTUD4 participates in the regulation of CSE-induced apoptosis by regulating the expression of PAI-1.

## Discussion

In this study, we demonstrated that, (i) cigarette smoke decreased OTUD4 in cellular and mouse models; (ii) OTUD4 silence promoted airway epithelia apoptosis induced by CSE, while OTUD4 reconstitution attenuated CSE induced apoptosis both in vivo and vitro; (iii) OTUD4 regulated airway epithelial cell apoptosis through modulating the ubiquitination and degradation of PAI-1 protein.

COPD is a heterogeneous disease, airway inflammation, oxidative stress and cell deaths forming network module in COPD pathogenesis. Regulated cell death (RCD) play an important role in physiological processes including maintenance of homeostasis. Excess RCDs causes airway inflammation, lung tissue injury and destruction in the pathogenesis and development of COPD [36]. Epithelial cells extending from the upper airways to the terminal alveoli are the first line of defense within the lungs. Dysregulated RCDs of airway epithelial cells including apoptosis, necroptosis, pyroptosis and ferroptosis promotes emphysema and lung destruction in COPD [37–40]. Here we also confirmed that airway epithelial cell apoptosis involved in the emphysematous destruction in COPD.

Ubiquitination proteasome system (UPS) is reported to be activated in COPD, with accumulation of ubiquitinated proteins in the lungs of severe COPD subjects [14, 15]. CSE treatment of airway epithelial cells, macrophages, and mouse lung tissue significantly increased histone deacetylase 2 (HDAC2) ubiquitination and degradation, which is related to steroid tolerance in COPD patients [41]. Thus, reconstruction of deubiquitinase USP17 blocked cigarette smoke extract induced destruction of HDAC2 [42]. The deubiquitylating enzyme USP25 is implicated to decreased under smoking, thus increasing bacteria load in lung epithelial cells [34]. In our research, we also found that de-ubiquitination enzyme OTUD4 decreased in CSE-treated bronchial epithelial cell and emphysema mouse model, which is suggested to involved in COPD pathogenesis. This finding might add a paradigm of that de-ubiquitination enzyme decreased under CSE treatment. Besides, this evidence supported the previous studies that cigarette smoke may stimulate the level of ubiquitination to involve in the development of COPD via increasing ubiquitin ligase and decreasing deubiquitinating enzymes.

As a deubiquitinate enzyme, OTUD4 was reported to regulating DNA damage repair, which play a role in modulating cell fate decision [20, 43]. Evidence showed that OTUD4 impaired DNA repair efficiency of non-small cell lung cancer (NSCLC) cells to regulate the cell cycle and cell apoptosis [43]. Besides, OTUD4 restrained the growth of various tumor including breast, liver, and lung cancer through activating tumor cell apoptosis [21]. Interestingly, knockdown of OTUD4 upregulated HEK239T and HeLa cell death and activated apoptotic makers [20]. In our study, we verified that OTUD4 silence increased airway epithelial cell apoptosis and restoration of OTUD4 attenuated the CSE induced epithelia apoptosis.

Although OTUD4 modulating cell death, the molecular mechanism of OTUD4 regulated cell apoptosis is still ambiguous. Our data indicated that OTUD4 is associated with PAI-1, a serine protease inhibitor (SERPIN), which is related to ubiquitin proteasome degradation in the regulating of cell death and was reported to promote angiogenesis and tumor cell survival by preventing apoptosis [44–49]. CSE induced ubiquitination and degradation of protein-promoted cell death of lung microvascular endothelial cells and lung fibroblasts [15] [34]. In addition, PAI-1 is reported to diminish the apoptosis of neutrophils by improving the expression of the antiapoptotic proteins [27]. The expression of PAI-1 is influenced by cigarette smoke and participated in the pathogenesis of lung diseases including COPD [50–52]. Previous study showed that PAI-1 is a downstream target that is negatively regulated by Ubiquitin-E3 Ligase RNF123, and PAI-1 knockdown reduced the proliferation and invasion of glioblastoma cell lines [48]. Likewise, we confirmed that PAI-1 is liable protein with a half-life of 3.3 hr and is regulated by ubiquitin-proteasomal mechanism.

OTUD4 executes its physiological functions mainly through the deubiquitinating enzyme activity to antagonize a K48- or K63-linked ubiquitination [16, 18]. Here, we found that OTUD4 interacts with PAI-1 and removes its K48-linked ubiquitination. Deactivating of PAI-1 in vivo and in vitro inhibit the protective effect of OTUD4 on cell apoptosis. Our data further revealed that OTUD4 regulates epithelial cell apoptosis through governing PAI-1 ubiquitin-proteasomal degradation. Above all, we infer that OTUD4 regulated CSE induced apoptosis is related to PAI-1 de-ubiquitination and degradation.

## Conclusion

In summary, the remarkable results of this study propose that overexpression of OTUD4 reduce cell apoptosis to protect against CSE treated lung epithelial cells and CSE mediated emphysematous model, which through PAI-1 activation. This compelling finding is helpful for understanding the pathogenesis of COPD and provide important implications on the clinical treatment of COPD in the future after further deep investigation.

### Supplementary information

original data files

Reproducibility Checklist

## Data Availability

The raw data acquired for the current study are available from the corresponding author upon reasonable request.
