# Supplementary material for: CSE reduces OTUD4 triggering lung epithelial cell apoptosis via PAI-1 degradation
Source: Cell Death Dis. 2023 Sep 19;14(9):614. doi: 10.1038/s41419-023-06131-1 (PMC10509146; doi:10.1038/s41419-023-06131-1)

Figure 1B

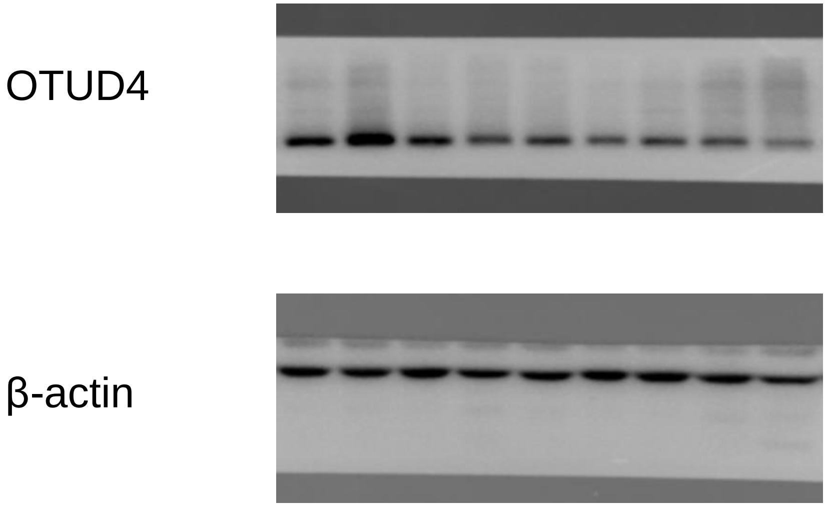

Figure 1D

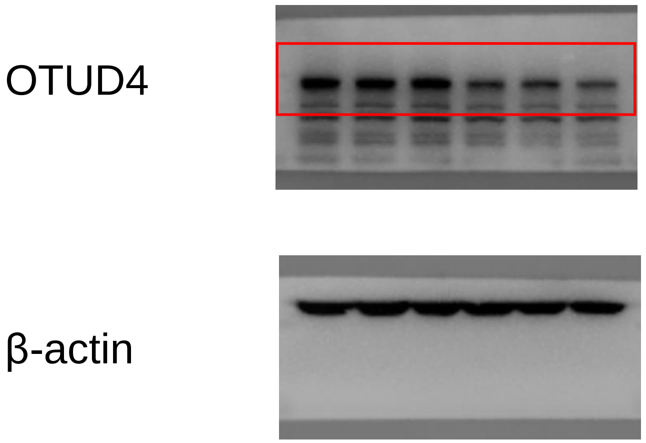

Figure 2B

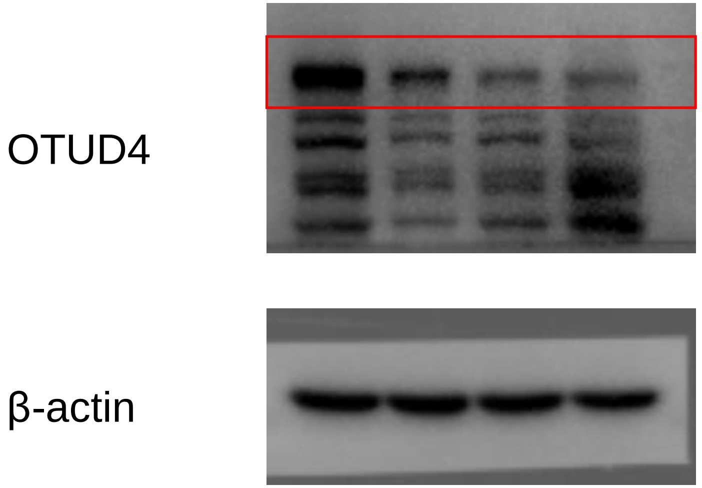

Figure 2E

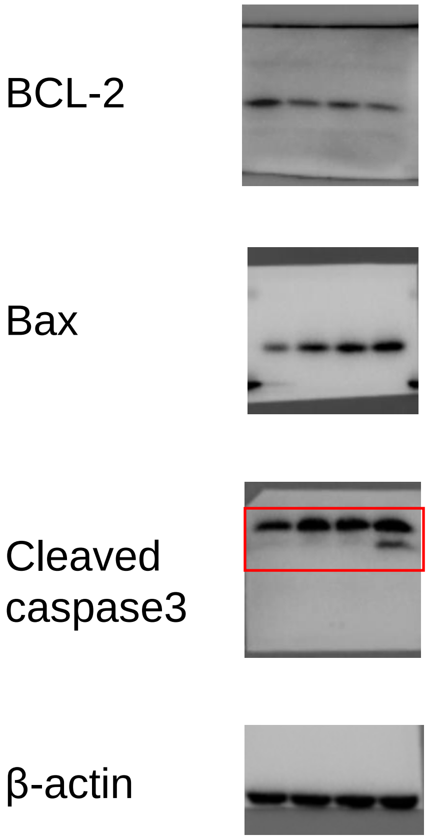

Figure 3A

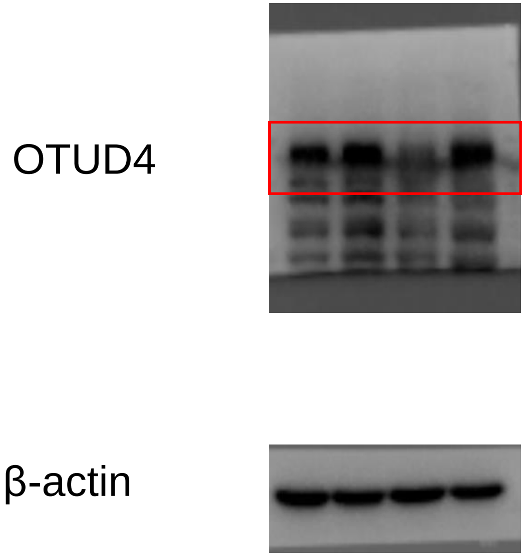

Figure 3D

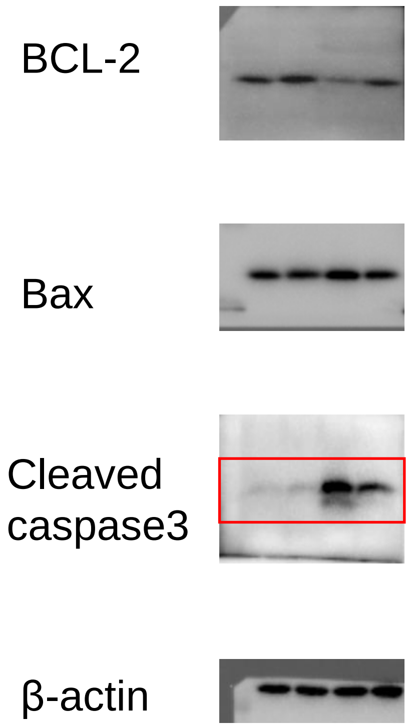

Figure 4A

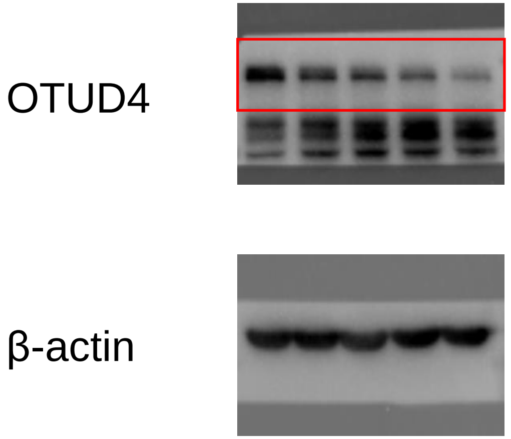

Figure 4B

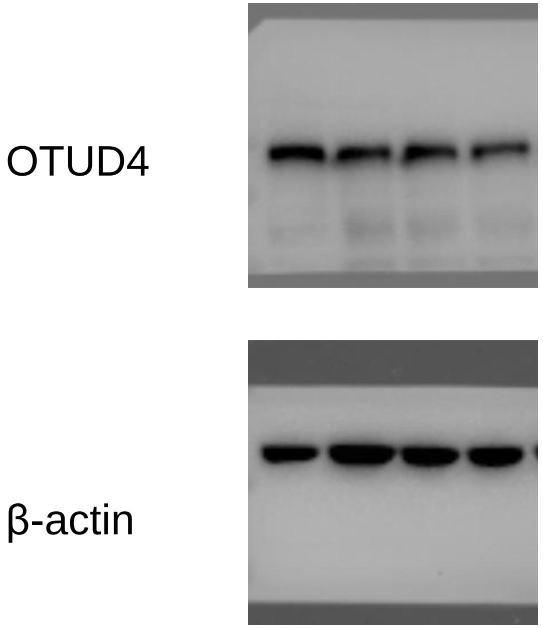

Figure 4C

OTUD4

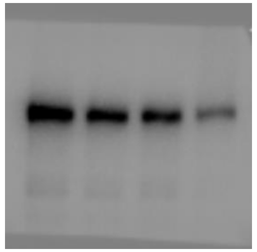

β-actin

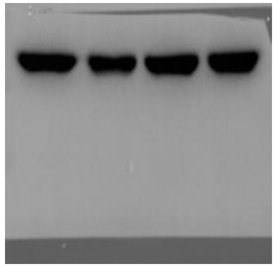

Figure 4D

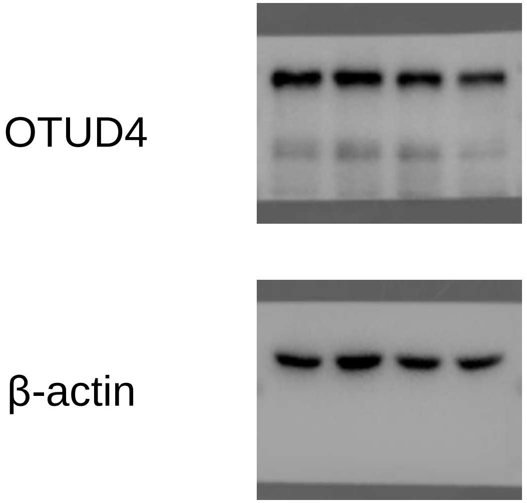

Figure 5B

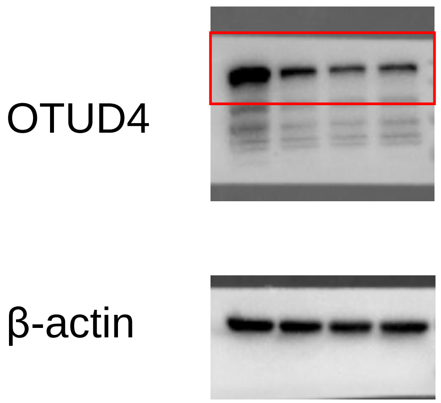

Figure 5C

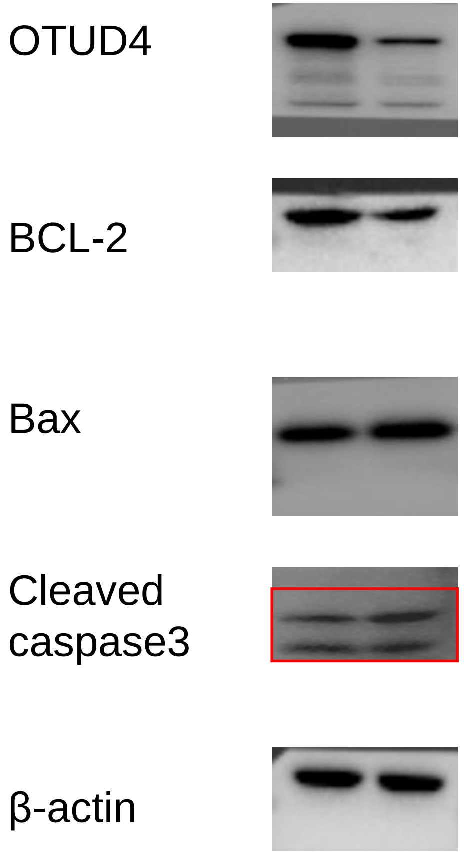

Figure 5E

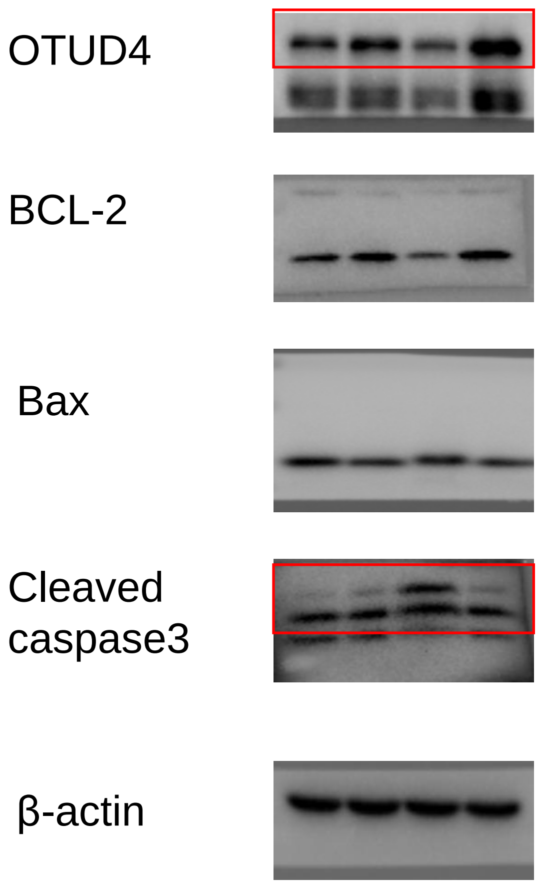

Figure 6A

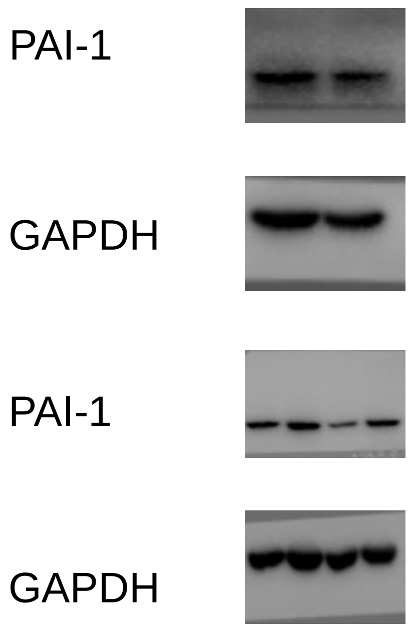

Figure 6B

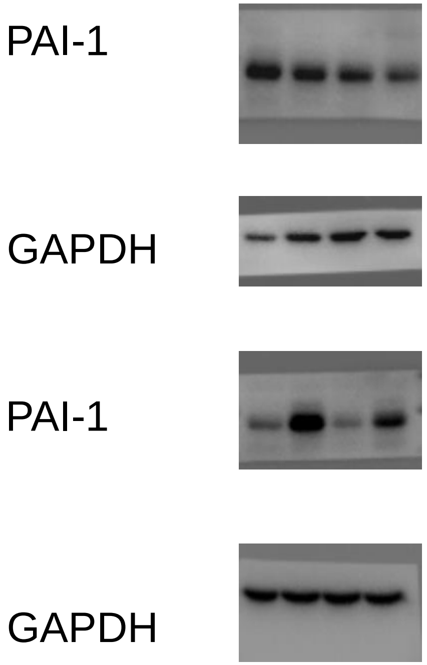

Figure 6C

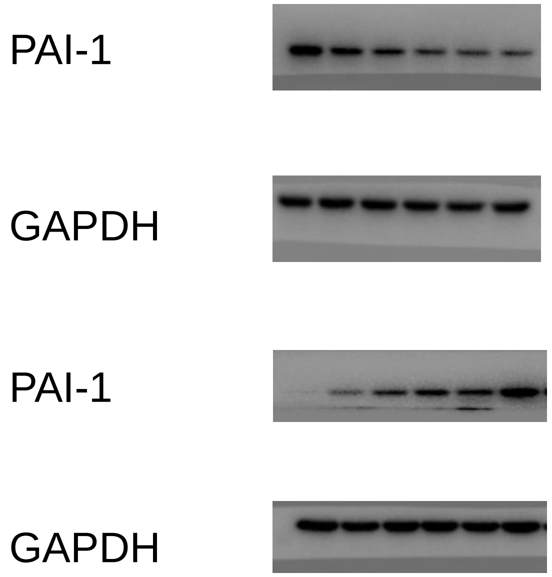

Figure 6D

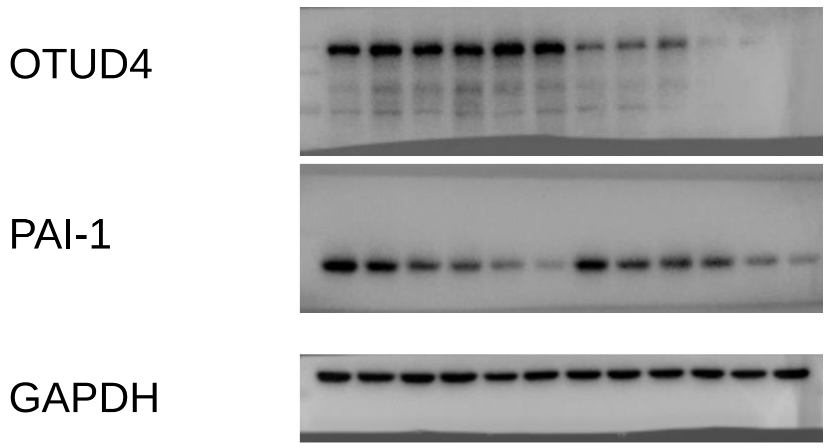

Figure 6E

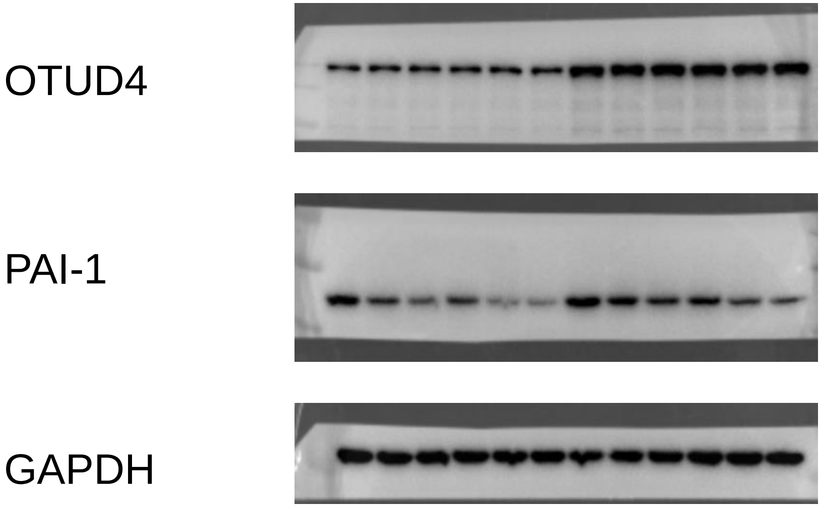

Figure 7A

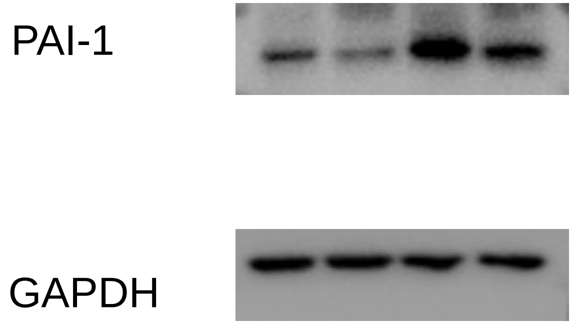

Figure 7B

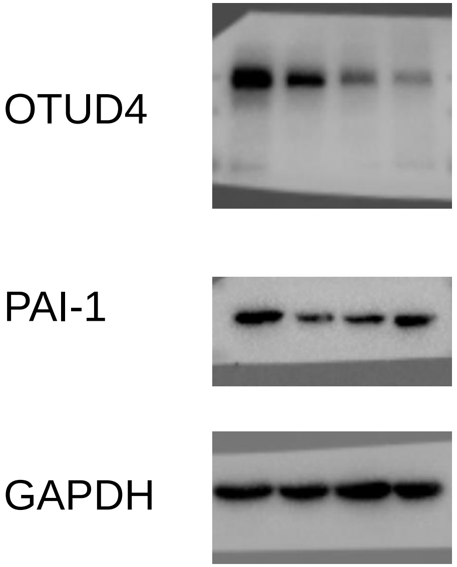

Figure 7D

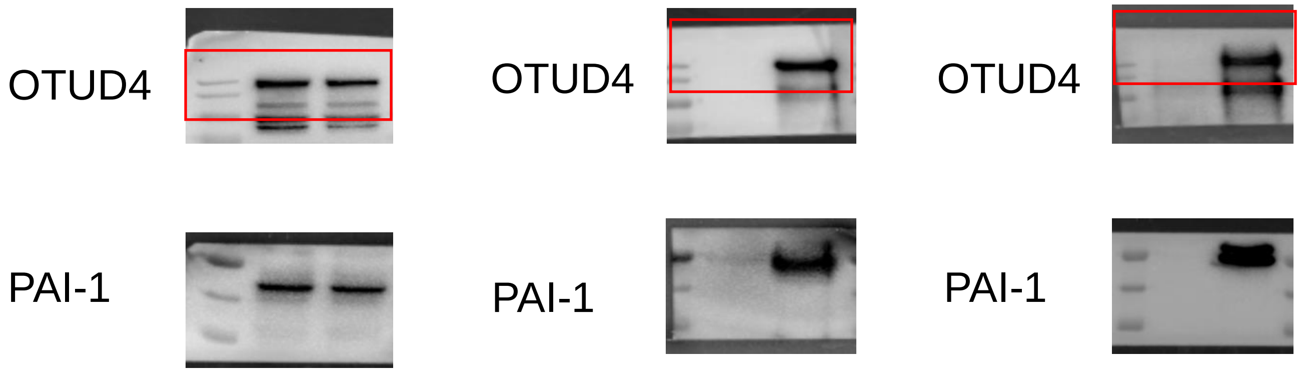

Figure 7E

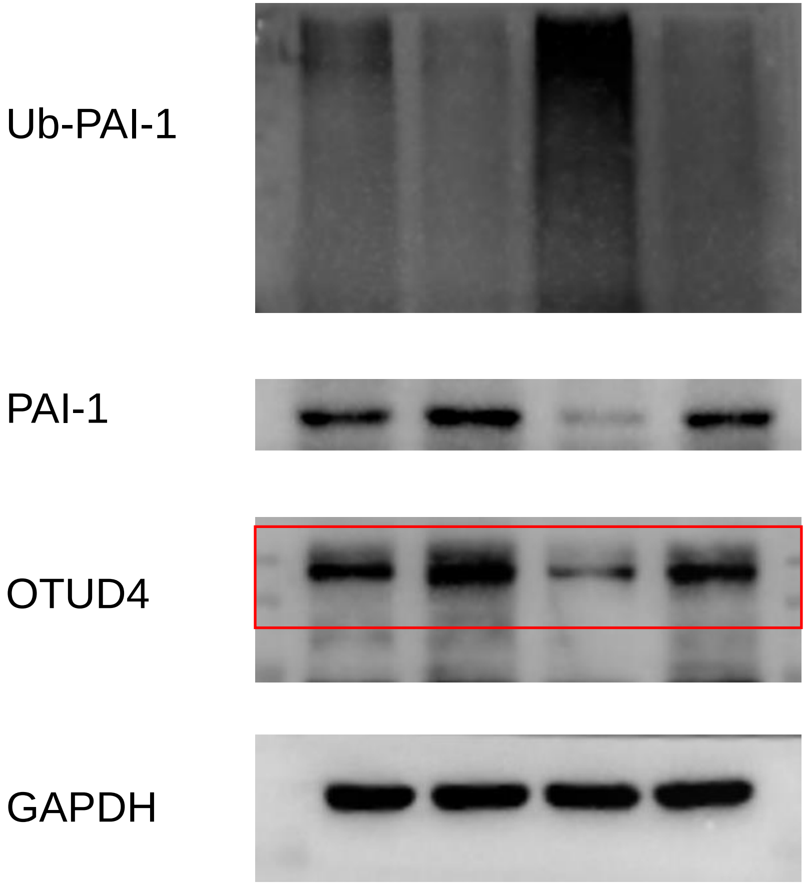

Figure 7F

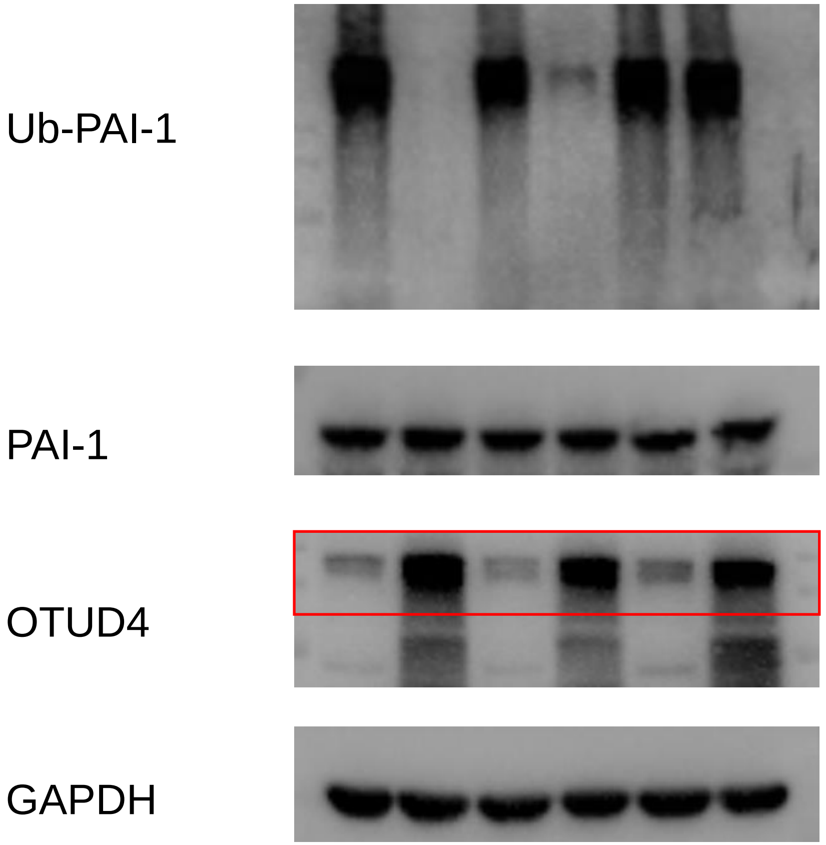

Figure 8A

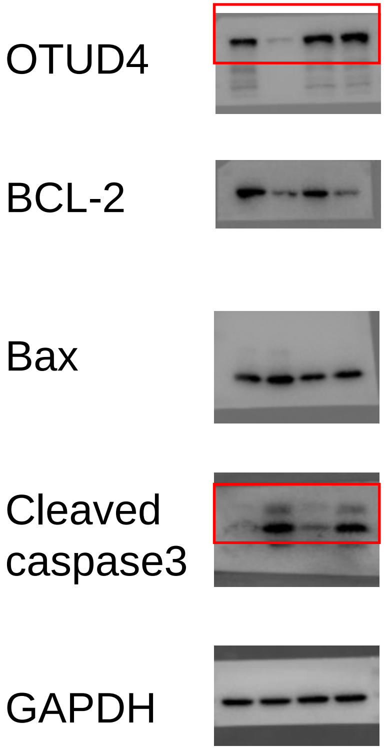

Figure 9E

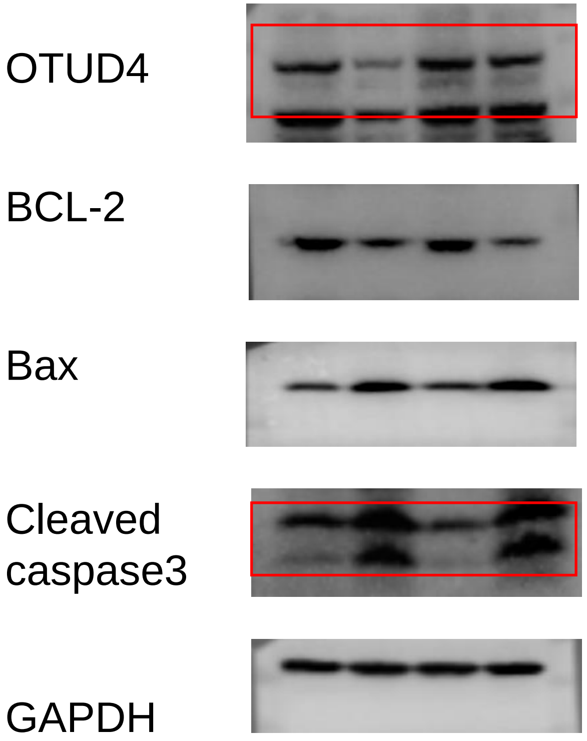

Supplement: Supplementary file 1 — original data files [file 41419_2023_6131_MOESM1_ESM.pdf]
